# QTL Mapping and RNA-Seq Revealed Loci and Candidate Genes Associated with Cold Tolerance of Wheat Root at Seedling Stage

**DOI:** 10.3390/genes17040447

**Published:** 2026-04-13

**Authors:** Xuanshang Liu, Chenyan Si, Jingwei Xi, Ling Qiao, Xingwei Zheng, Jiajia Zhao

**Affiliations:** 1 College of Agriculture, Shanxi Agricultural University, Jinzhong 030800, China; liuxuanshang0036@163.com (X.L.); scy990716@163.com (C.S.); xijingwei_1222@163.com (J.X.); 2 Institute of Wheat Research, Shanxi Agricultural University, Linfen 041000, China; qiaolingsmile@163.com (L.Q.); smilezxw@126.com (X.Z.)

**Keywords:** wheat, seedling stage, cold tolerance index, QTL mapping

## Abstract

Objectives: Low-temperature stress has become a key factor severely restricting seedling growth in wheat, highlighting the growing importance of research on low-temperature tolerance in wheat. Most previous studies focused on the aboveground organs, and only a minority specifically targeted the roots. Methods: In this study, a recombinant inbred line population derived from a cross between DH118 and Jinmai 919 was used to dissect the genetic regulatory mechanism underlying root growth and development in wheat seedlings under low-temperature conditions. Results: A total of 9 QTLs associated with low-temperature tolerance coefficient were identified; among them, *QCi.saw-2B* and *QCi.saw-5B* were validated to be significantly associated with cold tolerance in a natural population including 289 wheat accessions. Moreover, 13 candidate genes from QTL intervals regulating root growth and development under low-temperature stress were further identified based on RNA sequencing. Conclusions: These results provide a foundation for further exploring the regulatory mechanism of root growth and development in wheat under low-temperature stress.

## 1. Introduction

Wheat (*Triticum aestivum* L.) is a major staple crop, providing approximately 35% of dietary calories and protein for humans worldwide. Owing to its broad adaptability to diverse environmental conditions, it is essential to global food security [1]. Sown in autumn to overwinter as seedlings, winter wheat requires a long period of sustained low temperatures (winter chilling) to acquire the ability to flower in the following spring—a process known as vernalization [2]. In recent years, alongside global warming and the increasing frequency of extreme weather events, sudden temperature drops and prolonged frost during winter often cause severe damage to wheat seedlings, significantly impacting their growth and development and ultimately reducing final yields [3,4,5]. Breeding wheat varieties with strong cold tolerance is an effective measure to reduce frost damage [6]. Analyzing the mechanisms underlying low-temperature response is crucial for breeding cold-resistant varieties and developing cold-resistant cultivation techniques. Although numerous studies about wheat cold resistance have been published, most have focused on aboveground organs, with only a minority specifically targeting roots [7,8].

As a vital organ for absorbing water and nutrients from the soil, the root system performs functions such as anchorage, storage, uptake and transport and exhibits high developmental plasticity, enabling it to adapt relatively well to different environments [9]. Root growth, especially the fine roots which are crucial for water and mineral absorption, is generally more sensitive to temperature than that of aboveground plant parts [10]. Low temperatures prolong the cell cycle of root meristems, thereby inhibiting root growth, and this inhibitory effect becomes more pronounced as the duration of low temperatures increases [11]. Additionally, low temperatures reduce root length growth and biomass accumulation, leading to decreased root volume and surface area, which suppresses plant growth [12]. Under low-temperature stress, the phosphatidylcholine and phosphatidylethanolamine compositions in root zones of wheat typically increase to maintain membrane integrity [13]. The expression of genes related to auxin biosynthesis is suppressed, reducing auxin accumulation and thereby repressing the division potential of meristematic cells. This results in reduced meristem size and cell number, further suppressing root growth under low temperature [14].

To date, numerous quantitative trait loci (QTLs) for low-temperature tolerance have been detected across all 21 wheat chromosomes through linkage mapping and genome-wide association studies (GWAS) [15,16,17]. Båga et al. [18] identified QTLs for low-temperature tolerance on chromosomes 5A and 1D in two doubled haploid (DH) populations, with favorable alleles at both loci inherited from the cold-hardy parent Norstar. Sun et al. [19] developed a deep learning model FreezeNet to assess wheat freeze injury and identified 11 significant QTLs associated with freeze tolerance, four of which were validated to enhance freeze resistance in backcross progeny. Würschum et al. [20] identified 10 markers significantly associated with winter hardiness through GWAS, seven of which were located on chromosome 5A. Several loci identified on chromosome 5A are critical for freeze tolerance, as they contain key genes such as *CBF* and *VRN* [21]. The *Fr-A1* and *Fr-A2* loci are major contributors to frost tolerance on chromosome 5A. The *Fr-A2* locus harbors a cluster of *CBF* genes [22], which encode transcriptional factors that play important roles in activating low-temperature stress responses in plants [23]. Furthermore, copy number variations (CNVs) in the *CBF-A14* gene are essential for winter hardiness in wheat. *TaCBFA15*, *TaCBFA19*, *TaCBFA2* and *TaCBFD21.1* were found to be up-regulated under low temperatures, thereby enhancing freeze tolerance [24].

In addition, numerous studies have focused on other cold tolerance-related genes. NAC056 enhances freezing stress tolerance by regulating the expression of CBF pathway genes and also modulates root growth and nitrate assimilation under freezing conditions [25]. The wheat *SnRK2.8* gene is involved in responses to drought, salt, and cold injury and is strongly expressed in roots. Overexpression of *TaSnRK2.8* promotes root growth, reduces carbohydrate metabolism and soluble sugar content, and enhances cold stress tolerance in plants [26]. The MYB transcription factor family plays a vital role in plant responses to abiotic stresses. Overexpression of *TaMYB4* in wheat enhances the AsA-GSH cycle and up-regulates cold stress marker genes, thereby improving freeze tolerance in *Arabidopsis* [27]. Overexpression of *TaMYB56-B* in transgenic *Arabidopsis* results in higher survival rates after cold treatment, significantly enhancing freeze tolerance and inducing the up-regulation of *DREB1A/CBF3* and *COR15a* to improve cold adaptation [28]. Overexpression of protein kinase *TaABC1* reduces water loss, improves osmotic potential, photochemistry efficiency, and chlorophyll content, maintains cell membrane stability and regulates reactive oxygen species (ROS) homeostasis, thereby enhancing cold stress tolerance [29]. The *TaEXPA19* gene responds to cold stress with a differential expression pattern: rapid up-regulation in the aboveground part and delayed up-regulation in the underground part. Overexpression of *TaEXPA19* significantly promotes root growth and enhances cold tolerance in transgenic plants [30]. In wheat roots, the *TaEXPA8* gene exhibits increased expression under cold conditions. Overexpression of *TaEXPA8* in *Arabidopsis* improves cold tolerance by enhancing antioxidant activity [31]. *TaEXPB7-B*, a β-extending protein gene, is induced by low temperature and ABA and regulated through the ABA-TaWABI5 pathway; its overexpression promotes plant growth and enhances cold resistance by increasing antioxidant enzyme activity and osmotic regulation capacity [32].

This study aimed to investigate the effects of low-temperature stress on root development at the seedling stage of wheat under different cultivation regimes and to explore genetic loci and related candidate genes with cold tolerance. The results indicated that low temperatures significantly inhibited root growth of wheat seedlings. with the inhibitory effect varying across different cultivation regimes. In this study, nine QTLs associated with cold tolerance index were identified. Combined with transcriptome data, 13 candidate genes regulating root development under low temperature were screened, 7 of which were validated using the KASP assay in a natural population.

## 2. Materials and Methods

### 2.1. Plant Materials

A F_10_ recombinant inbred line (RIL) population consisting of 165 lines derived from a cross between DH118 and Jinmai 919 was used for quantitative trait locus (QTL) mapping. The DH118 was a high-yielding cultivar selected for irrigated conditions, with strong cold resistance, and Jinmai 919 was a dryland cultivar with moderate cold resistance. A natural population was used for QTL effect verification, consisted of 289 wheat germplasms collected from Shanxi Province, China, including 127 irrigated cultivars, 122 dryland cultivars and 40 landraces (Table A1).

### 2.2. Seedling Material Culture

The low-temperature experiment was arranged in a completely randomized design with three biological replicates. The experiment was conducted with two culture regimes: vermiculite cultivation (VC) and nutrient solution culture (NS). For each substrate, two temperature treatments were set at 6 °C and 24 °C.

Healthy and uniform-sized seeds free of pests and diseases were selected. For each line (variety), 20 seeds were rinsed with distilled water, soaked in 3% H_2_O_2_ for 3 min, and then washed three times with distilled water. The sterilized seeds were then placed in Petri dishes with one sheet of filter paper moistened with 5 mL of distilled water and incubated in darkness at 22 °C until germination.

Healthy, disease-free seeds with uniform growth vigor were transplanted into pots filled with a mixture of vermiculite and Hoagland nutrient solution for vermiculite cultivation. The pots were 9.2 cm in height, with a bottom diameter of 4.7 cm and a top diameter of 7 cm. The pots were then placed in a growth chamber maintained at 24 °C with a 16 h light/8 h dark photoperiod and 60% relative humidity and were watered regularly throughout the cultivation period. Nutrient solution culture was performed in hydroponic boxes filled with Hoagland nutrient solution. The boxes were 70 cm long, 45 cm wide, and 35 cm high. The hydroponic boxes were covered with perforated panels containing 1 cm diameter holes, with five seedlings per hole. Temperature and photoperiod were kept consistent with those in the vermiculite cultivation system, and the nutrient solution was renewed timely. After 5 days of culture, the wheat seedlings were transferred to two temperature regimes for a 5-day treatment. Hoagland nutrient solution with a pH of 5.8–6.0 was used in the experiment [33].

### 2.3. Root Phenotyping of Seedlings

Roots and leaves of wheat seedlings were severed at their junction, rinsed with distilled water, and then root images of three seedlings were captured using a Microtek Scanmakeri 800 plus scanner. The total root length (TRL), maximum root length (MRL), root average diameter (RD), root surface area (RA) and root volume (RV) were measured using a LA-S Plant Root Analysis System (Wseen, Hangzhou, China).

### 2.4. Measurement of Embryo Size

To determine the relationship between embryo size and cold tolerance, cold-tolerant and cold-sensitive lines were selected based on the phenotypic data from the seedling treatment groups. Healthy, uniformly sized and plump seeds were chosen for embryo size measurement. Ten seeds were used per replicate, with three biological replicates in total, for subsequent cold tolerance evaluation.

### 2.5. Statistical Analysis

Data were processed using Microsoft Excel 2007 and IBM SPSS Statistics 27 software. Cold tolerance index (CI), mean values, standard deviation (SD), coefficients of variation (CV), and correlations among different indicators were calculated. Correlation heatmaps were generated using Origin 10.2 software.

### 2.6. Genetic Linkage Map Construction and QTL Analysis

Genomic DNA was extracted using the modified CTAB method [34]. Genotyping was subsequently performed. The high-density genetic linkage map was constructed according to the method described by Xu et al. [35]. QTL mapping for maximum root length (MRL), total root length (TRL), average root diameter (RD), root surface area (RA), root volume (RV), and cold tolerance coefficient (CI) of wheat was performed using the composite interval mapping (CIM) method implemented in WinQTLCart 2.5. The minimal LOD score to accept the presence of a QTL was set at 2.5. We consider QTLs separated by less than 1 cM or sharing identical flanking markers as the same locus and name the identified QTLs following the method of McCouch et al. [36]. Finally, the flanking marker sequences of the mapped QTLs and those of previously reported similar loci were aligned against the Chinese Spring reference genome sequence using the WheatOmics multi-omics database (http://wheatomics.sdau.edu.cn, accessed on 10 December 2025) [37].

### 2.7. RNA Extraction and Transcriptome Analysis

Root transcriptome sequencing was performed on the low-temperature-sensitive line R9 and low-temperature-tolerant line R130 selected from the RIL population. Total RNA from roots of wheat seedlings in both the treatment and control groups was extracted and purified using the Quick RNA Isolation Kit (Beijing Huayueyang Biotechnology Co., Ltd., Beijing, China); three biological replicates were set for each sample. The specific experimental steps were performed strictly in accordance with the kit instructions.

The extracted total RNA from the plants was sent to Beijing Novogene Co., Ltd. (Beijing, China) for cDNA library construction and transcriptome sequencing. We performed quality control and filtering on the raw sequencing data, which were then used for subsequent analyses. Differentially expressed genes were screened based on *padj* ≤ 0.05. GO functional enrichment analysis and KEGG pathway enrichment analysis were performed on the differentially expressed gene set using the clusterProfiler 4.0 software package.

### 2.8. KASP Marker Development, Validation and Candidate Gene Prediction

Based on the QTL mapping results, SNP markers closely linked to the major QTL were selected for KASP marker development [38]. Using the probe sequences, two allele-specific primers (F1, F2) and one common reverse primer (R) were designed for each locus. A FAM fluorescent tag sequence (5′-GAAGGTGACCAAGTTCATGCT-3′) was added to the 5′ end of the allele-specific primer F1, and a HEX fluorescent tag sequence (5′-GAAGGTCGGAGTCAACGGATT-3′) was added to the 5′ end of primer F2.

The KASP primers were designed using the PolyMarker online tool (http://www.polymarker.info/) and synthesized by Sangon Biotech Co., Ltd. (Shanghai, China). The developed KASP markers were used to genotype 289 wheat lines from Shanxi Province, so as to verify the genetic effect of the target candidate gene (Table A2 and Table A3).

Target genes within the functional interval of the QTL were identified using the JBrowse platform on the WheatOmics website (http://wheatomics.sdau.edu.cn, accessed on 10 December 2025) [39]. Based on the Gene Ontology (GO) database, genes within the target region were functionally annotated and subjected to enrichment analysis, and genes associated with root cold tolerance were selected for further study. Cis-acting elements in the promoter regions of the candidate genes were predicted using the PlantCARE database (https://bioinformatics.psb.ugent.be/webtools/plantcare/html/, accessed on 5 April 2026). For phylogenetic analysis, MEGA 7 software was employed to construct phylogenetic trees for the candidate genes and their homologous genes isolated from *Zea mays*, *Oryza sativa* and other plant species. Finally, the obtained data were visualized using TBtools-II v2.466 and the iTOL v7 online tool (https://itol.embl.de/).

## 3. Results

### 3.1. The Effects of Low-Temperature Stress on Root Growth of Wheat at the Seedling Stage

To investigate the effects of low temperature on root growth in wheat seedlings, two cultivation conditions were established to simulate seedling growth environments. Following low-temperature treatment, root growth of wheat seedlings was significantly inhibited, and the inhibitory effects differed between the two culture conditions. Under nutrient solution culture (NS), the maximum root length (MRL), total root length (TRL), root surface area (RA) and root volume (RV) decreased by 54.01%, 56.31%, 49.40%, and 50.00%, respectively. Under vermiculite cultivation (VC), MRL decreased by 20.73%, TRL by 35.01%, RA by 33.54%, and RV by 33.33%. Under both cultivation conditions, low temperature increased the average root diameter (RD) at the seedling stage. It increased by 7.32% under NS and by 14.29% under VC. Overall, TRL showed the largest reduction under low temperature and was thus more severely affected (Table 1).

Low temperature inhibited root growth of wheat at the seedling stage under both environments, and the reduction magnitude was larger under NS than under VC. With increasing temperature, similar trends were observed under both culture conditions: TRL, MRL, RA, and RV all increased, while RD decreased by 7.32% under NS and 14.29% under VC (Table 1).

**Figure 1:**
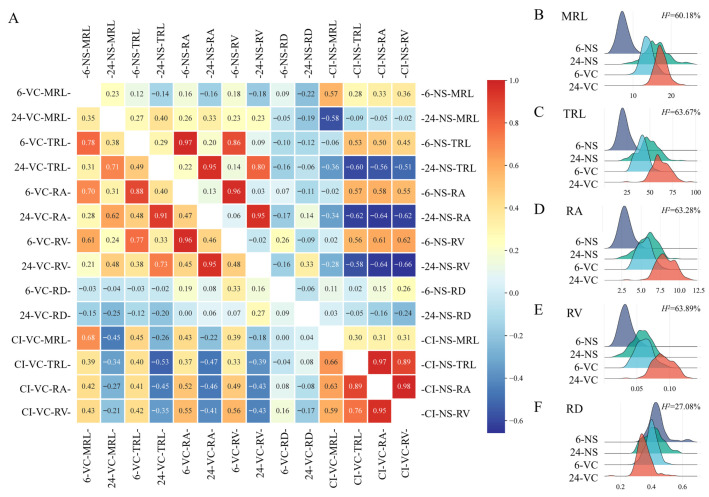
Correlation heatmap of wheat seedling root traits (**A**). Ridge plot of seedling root traits (**B**–**F**).

The correlation heatmap showed that TRL and RA were significantly positively correlated with RV at low temperature under both cultivation conditions, and the same trend was observed with increasing temperature. The cold tolerance index of TRL, RD, and RA was also significantly positively correlated under the two cultivation conditions. At 24 °C, MRL, TRL, RA and RV were negatively correlated with the cold index under both culture conditions. Overall, low temperature exhibited a stronger inhibitory effect on root growth under NS conditions. For the five seedling root traits, the *H*^2^ values ranged from 0.6018 to 0.6389, except for RD, indicating that these traits were strongly influenced by genetic factors (Figure 1).

### 3.2. Effect of Seed Embryo Size on Root Length of Wheat Seedlings Under Low Temperature

**Figure 2:**
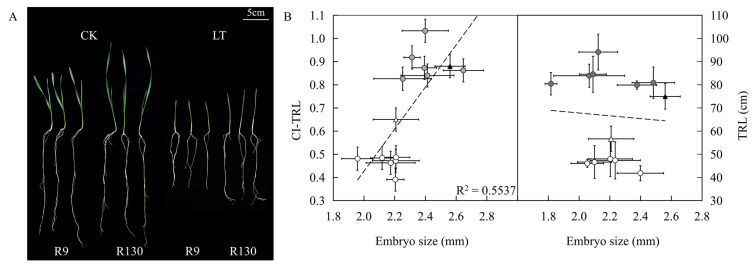
Morphological differences at seedling stage of R9 and R130 (**A**). Relationship between embryo size, CI, and TRL in RILs under low temperature (**B**). Shaded circles: cold-resistant lines; open circles: cold-sensitive lines; black triangle: DH118; open triangle: Jinmai 919. The dotted line represents the trend line fitted based on the scatter points.

Embryo measurements were performed on the selected cold-resistant and cold-sensitive lines according to the CI values. The results demonstrated that DH118, a parental line of the RIL population, had larger embryos, longer total root length under both low-temperature and normal conditions, and a higher CI under low-temperature stress compared with Jinmai 919. Analysis of the relationship between low-temperature coefficient and embryo size under low-temperature conditions revealed that the average embryo size of cold-resistant lines at the upper tail (2.40 mm) was significantly larger than that of cold-sensitive lines at the lower tail (2.17 mm); when embryo size was similar (average embryo size: 2.16 mm in cold-resistant lines vs. 2.20 mm in cold-sensitive lines), the cold-resistant lines still exhibited a greater total root length (Figure 2).

### 3.3. QTL Mapping

In this study, QTL mapping was performed for five root traits at the seedling stage in wheat using a RIL population. A total of 37 QTLs associated with root-related traits under low-temperature stress were detected. Of these, 9 QTLs were associated with TRL, 10 with MRL, 4 with RD, 4 with RA and 10 with RV. Furthermore, 9 QTLs related to cold tolerance index were also detected. These QTLs were distributed on 16 chromosomes except for 1D, 4D, 5D, 6A and 7A. The phenotypic variation explained (PVE) by these QTLs ranged from 5.94% to 29.92% (Table 2).

For TRL, nine QTLs were identified on chromosomes 1A, 2B, 3A, 4B, 5A, 5B, 6D, 7B and 7D, with PVE ranging from 5.94% to 29.92%. Among these, *QTrl.saw-4B* and *QTrl.saw-5A* were detected across two environments and thus considered stable QTLs.

For MRL, 10 QTLs were identified on chromosomes 2A, 3B, 4B, 5A, 5B, 6B, 7B and 7D, with PVE ranging from 6.31% to 14.11% across environments. *QMrl.saw-5A*, detected in two environments, was characterized as a stable QTL.

For RA, four QTLs were detected on chromosomes 2B, 3A, 3B and 5A, with PVE ranging from 8.71% to 19.12%. Among them, *QRa.saw-5A* was identified across two environments.

For RV, a total of 10 QTLs were mapped on 9 chromosomes including 1B, 2B, 2D, 3A, 3B, 3D, 5A, 6B and 6D. These QTLs explained 6.49% to 16.68% of the phenotypic variation individually. Among them, *QRv.saw-5A* was detected in two environments and identified as a stably expressed QTL.

For RD, four QTLs were detected on chromosomes 3A, 3B, 6D and 7D, with PVE ranging from 6.89% to 14.28% across environments.

**Figure A1:**
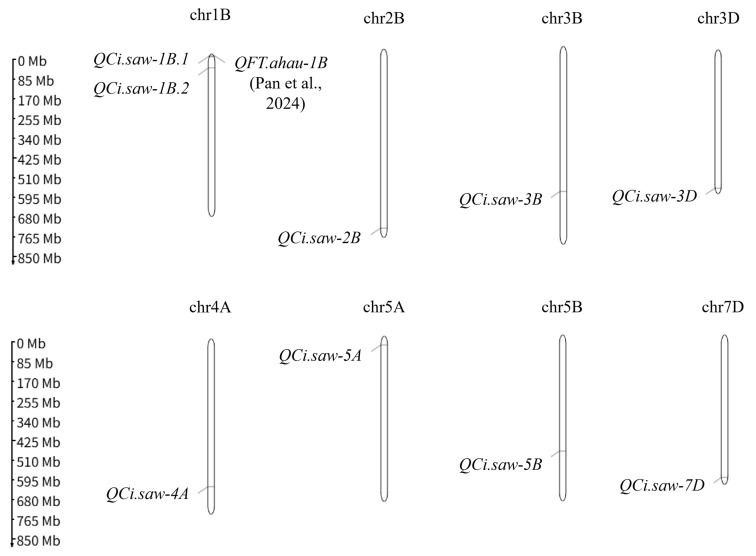
Physical positions of QTLs for CI [6].

QTL mapping for the cold tolerance index was performed using the RIL population. A total of 9 QTLs were detected on chromosomes 1B, 2B, 3B, 3D, 4A, 5A, 5B and 7D, namely *QCi.saw-1B.1*, *QCi.saw-1B.2*, *QCi.saw-2B*, *QCi.saw-3B*, *QCi.saw-3D*, *QCi.saw-4A*, *QCi.saw-5A*, *QCi.saw-5B* and *QCi.saw-7D*. The PVE by these QTLs ranged from 6.13% to 13.94%, and four of them were consistently detected across two environments (Table 3; Figure A1). Three of these QTLs (*QCi.saw-1B.1*, *QCi.saw-1B.2* and *QCi.saw-5B*) have been reported in previous studies [6,15,40].

Among these QTL regions, we identified nine overlapping QTLs for root traits at the seedling stage in wheat, suggesting that these loci collectively regulate root development in wheat seedlings from different aspects. For example, two genomic regions, *QTrl.saw-3A/QRa.saw-3A/QRv.saw-3A* and *QTrl.saw-5A/QRa.saw-5A/QRv.saw-5A*, collectively control TRL, RA and RV. *QMrl.saw-5B.1/QTrl.saw-5B/QCi.saw-5B* and *QMrl.saw-7D/QTrl.saw-7D/QCi.saw-7D* coordinately regulate MRL, TRL and CI. Furthermore, *QTrl.saw-2B*, *QRa.saw-2B* and *QCi.saw-2B* were co-localized in the interval 738.69–781.58 cM. Similarly, *QRv.saw-3B*, *QRd.saw-3B* and *QRa.saw-3B* were mapped simultaneously in the interval 389.04–561.09 cM.

### 3.4. QTL Additive Effect Analysis

**Figure 3:**
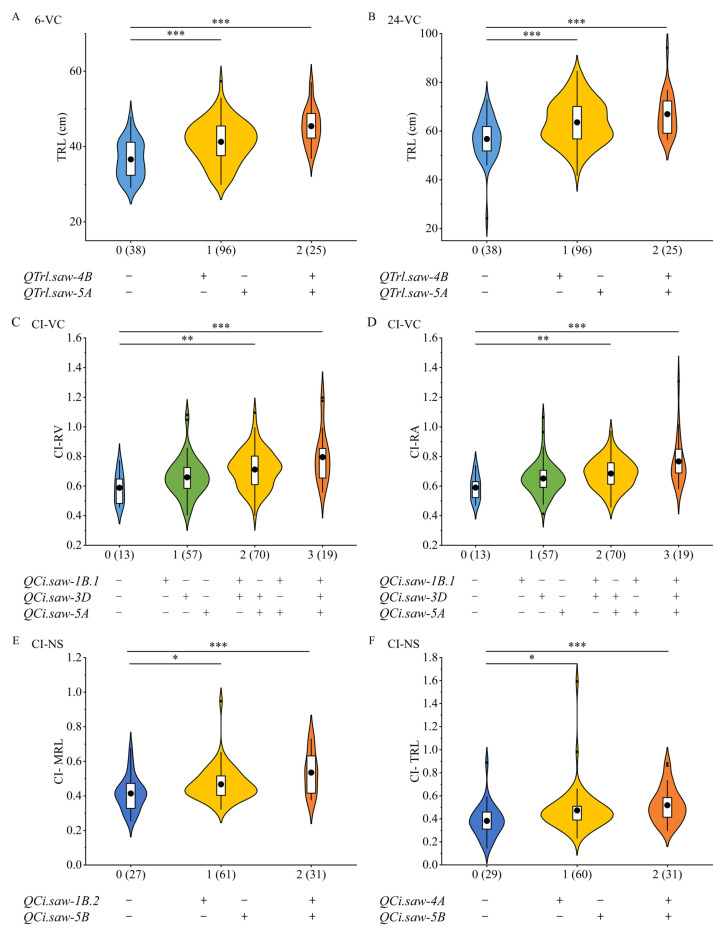
Analysis of the additive effects of stable QTLs. (**A**,**B**) Additive effect analysis of QTLs for TRL; (**C**,**D**) Additive effect analysis of QTLs for cold tolerance index under VC condition; (**E**,**F**) Additive effect analysis of QTLs for cold tolerance index under NS condition. + and − indicate the favorable and unfavorable alleles for the certain locus, respectively. *, ** and *** indicate significant differences at the 0.05, 0.01 and 0.001 probability levels, respectively.

In general, the accumulation of favorable alleles has a positive effect on root growth in wheat seedlings. In this study, a total of 5 stable QTLs for seedling root traits were identified, including 2 QTLs for TRL (*QTrl.saw-4B* and *QTrl.saw-5A*), 1 QTL for MRL (*QMrl.saw-5A*), 1 QTL for RA (*QRa.saw-5A*), and 1 QTL for RV (*QRv.saw-5A*). In addition, nine QTLs associated with cold tolerance index were detected, among which *QCi.saw-1B.1*, *QCi.saw-3D* and *QCi.saw-5A* were identified across multiple environments. *QCi.saw-1B.2* and *QCi.saw-4A* were significantly associated with MRL and TRL, respectively, while *QCi.saw-5B* was identified as a pleiotropic QTL that simultaneously regulated both root traits. Additive effect analysis of stable loci for TRL and CI indicated that an increased number of favorable alleles contributed to better tolerance to low-temperature stress in plants (Figure 3).

### 3.5. Validation of QTLs in a Natural Population

Six KASP markers for CI were developed based on the QTLs identified by linkage analysis (Table A3). These QTLs were validated using a natural population composed of 289 wheat accessions from Shanxi Province, China. The results indicated that *QCi.saw-2B* and *QCi.saw-5B* were significantly associated with the target traits.

**Figure 4:**
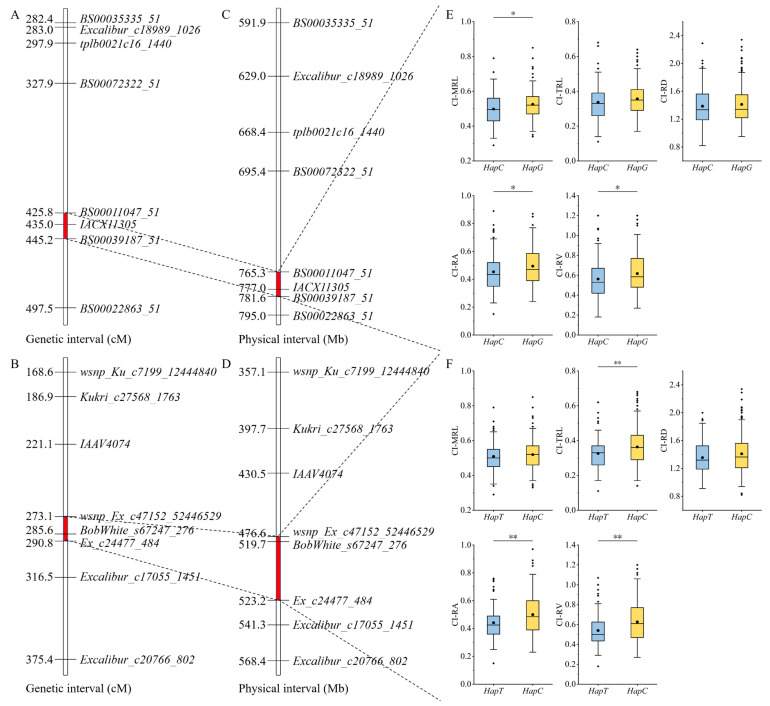
Genetic interval of *QCi.saw-2B* and *QCi.saw-5B* (**A**,**B**). Physical interval of *QCi.saw-2B* and *QCi.saw-5B* (**C**,**D**). Effect analysis of *QCi.saw-2B* and *QCi.saw-5B* in the RIL population (**E**,**F**). * indicates a significant difference at the 0.05 probability level. ** indicates a significant difference at the 0.01 probability level.

Significant differences were detected at *QCi.saw-2B* for CI of maximum root length, root surface area, and root volume. Significant differences were detected at *QCi.saw-5B* for CI of total root length, root surface area and root volume (Figure 4).

### 3.6. Analysis of Transcriptome Sequencing Results

Root samples of the cold-resistant line R130 (CR) and the cold-sensitive line R9 (CS) under low-temperature (LT) and control (CK) conditions were subjected to sequencing. A total of 129.98 Gb of clean data were generated, with at least 9.46 Gb of clean data obtained for each sample. The Q20 ratio exceeded 99.00%, the Q30 ratio was above 97.00%, and the average GC content was 51.30%. The data were of qualified quality and high accuracy and thus were suitable for subsequent analysis (Table A4).

**Figure 5:**
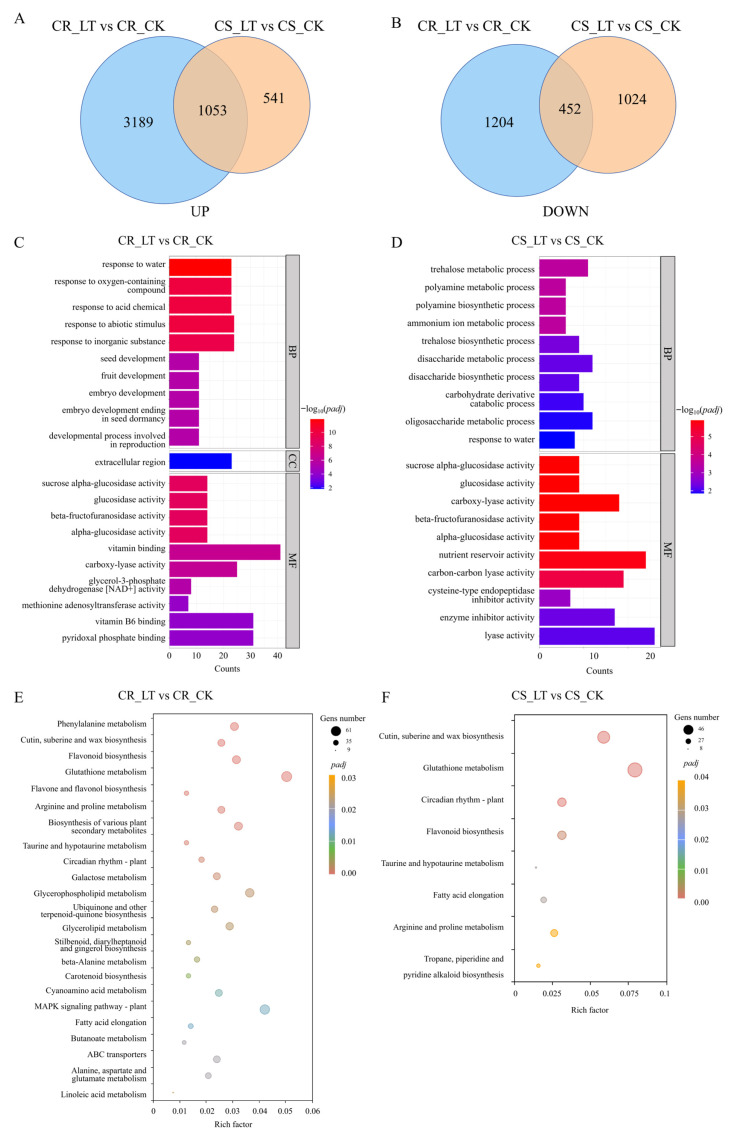
Venn diagram of differentially expressed genes (**A**,**B**). GO enrichment analysis (**C**,**D**). KEGG annotation analysis (**E**,**F**).

Following treatment, the gene expression patterns of the two lines were altered. The CR line exhibited substantial changes in its expression profile after low-temperature treatment compared with the control. In contrast, the CS line showed only minor changes in gene expression patterns following treatment (Figure 5A,B).

We subsequently analyzed the differentially expressed genes (DEGs) in the two lines with contrasting low-temperature tolerance. A total of 5898 DEGs were identified in the CR line between low-temperature and normal conditions, among which 4242 genes were up-regulated and 1656 genes were down-regulated. For the CS line, a total of 3070 DEGs were detected between low-temperature and normal conditions, among which 1594 genes were up-regulated and 1476 genes were down-regulated. A total of 1053 genes were commonly up-regulated and 452 genes were commonly down-regulated in response to low temperature in both CR and CS lines. Low-temperature stress altered gene expression in wheat seedling roots, with more genes up-regulated than down-regulated. The two varieties differing in low-temperature tolerance exhibited distinct responses to low-temperature stress at the transcriptome level.

Based on GO enrichment analysis, the DEGs between low-temperature and normal conditions in the CR line and CS line were analyzed; a total of 1121 DEGs were annotated in CR in the LT vs. CK comparison, among which 509 DEGs were significantly enriched in 88 GO categories (*padj* ≤ 0.05). In the biological process (BP) category, 43 terms were significantly enriched, including response to water (GO:0009415), response to abiotic stimulus (GO:0009628), and response to acidic chemicals (GO:0001101). In the cellular component (CC) category, genes were significantly enriched in the extracellular region (GO:0005576). In the molecular function (MF) category, 44 terms were enriched, including beta-fructofuranosidase activity (GO:0004564), sucrose alpha-glucosidase activity (GO:0004575), and glucosidase activity (GO:0015926) (Figure 5C).

A total of 505 DEGs were annotated in the CS in the LT vs. CK comparison, among which 152 DEGs were significantly enriched in 57 GO categories. In the biological process (BP) category, 33 GO terms were significantly enriched, including trehalose metabolic process (GO:0005991), polyamine metabolic process (GO:0006595), and polyamine biosynthetic process (GO:0006596). In the molecular function (MF) category, 24 terms were significantly enriched, such as carboxy-lyase activity (GO:0016831), beta-fructofuranosidase activity (GO:0004564), and sucrose alpha-glucosidase activity (GO:0004575) (Figure 5D).

A total of 98 significant DEGs were annotated between the CS and CR, among which 88 DEGs were up-regulated and 10 DEGs were down-regulated. These DEGs were significantly enriched in 45 GO terms, including biological processes such as metabolism and biosynthesis, developmental response, and enzyme activity regulation.

To further explore the biological functions of DEGs, enrichment analysis was performed based on the KEGG database to identify the major metabolic and signal transduction pathways in which DEGs are involved. In the CR line, a total of 518 DEGs were identified, which were significantly enriched in 23 metabolic pathways; these DEGs exhibited pronounced changes in pathways related to energy metabolism, biosynthesis of secondary metabolites, and maintenance of plant homeostasis. In contrast, the CS line exhibited 147 DEGs that were significantly enriched in eight metabolic pathways, with remarkable alterations mainly observed in pathways involved in plant homeostasis maintenance and energy metabolism. Among them, seven significantly enriched metabolic pathways were shared by the two lines, namely glutathione metabolism, cutin, suberine and wax biosynthesis, plant circadian rhythm, flavonoid biosynthesis, taurine and hypotaurine metabolism, fatty acid elongation, and arginine and proline metabolism (Figure 5E,F).

The cold-resistant line R130 and the cold-sensitive line R9 displayed distinct differences in their metabolic pathways. Notably, low-temperature stress significantly affected pathways related to energy metabolism, secondary metabolite biosynthesis, amino acid biosynthesis, and the plant MAPK signaling pathway in wheat seedlings. Moreover, the tolerant variety R130 exhibited more extensive metabolic pathway reprogramming, conferring enhanced low-temperature stress tolerance.

### 3.7. Prediction of Candidate Genes Associated with Cold in Roots

**Figure 6:**
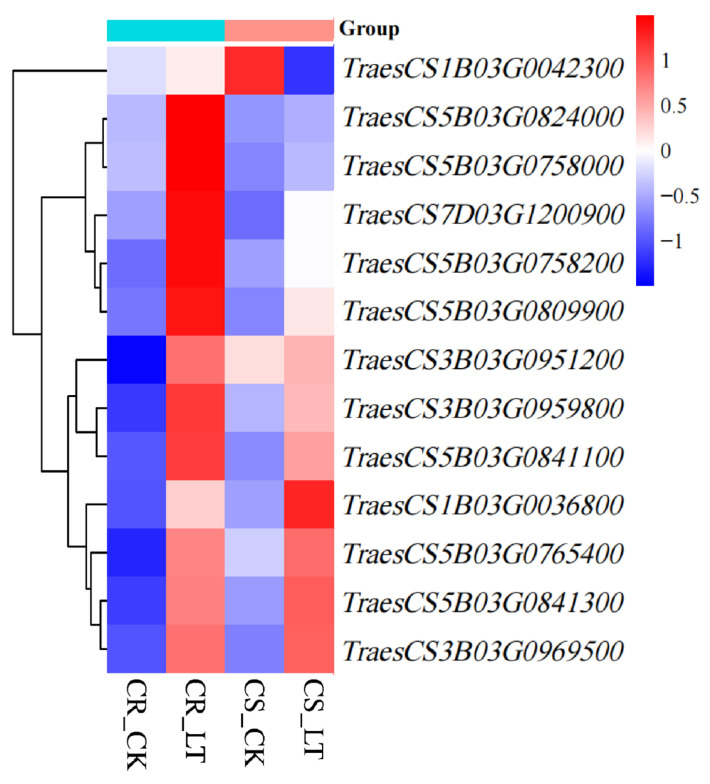
Heatmap of expression levels of candidate genes responding to low-temperature stress.

Nine QTLs associated with the low-temperature tolerance coefficient were mapped via linkage analysis, and transcriptomic profiling identified 68 DEGs (48 from CR line, 20 from CS line, Table A5 and Table A6) located within the genomic regions of these QTLs. By combining GO functional and KEGG pathway enrichment analyses, 13 pathway-enriched DEGs were identified, including WRKY transcription factor family member *TraesCS3B03G0951200*, PRR family member *TraesCS5B03G0809900*, BAG protein family member *TraesCS3B03G0959800*, ABC transporter family member *TraesCS3B03G0969500*, DTX40 gene *TraesCS5B03G0824000*, and RBOH protein gene *TraesCS5B03G0765400*. Additionally, seven enzyme-encoding genes were identified, namely *TraesCS1B03G0036800* (encoding methionine S-methyltransferase), *TraesCS5B03G0841300* (encoding ornithine decarboxylase), *TraesCS1B03G0042300* (encoding glutathione S-transferase), *TraesCS5B03G0841100* (encoding phospholipase), *TraesCS7D03G1200900* (encoding sucrose phosphate synthase), and two β-glucosidase-encoding genes, *TraesCS5B03G0758000* and *TraesCS5B03G0758200* (Figure 6).

**Figure 7:**
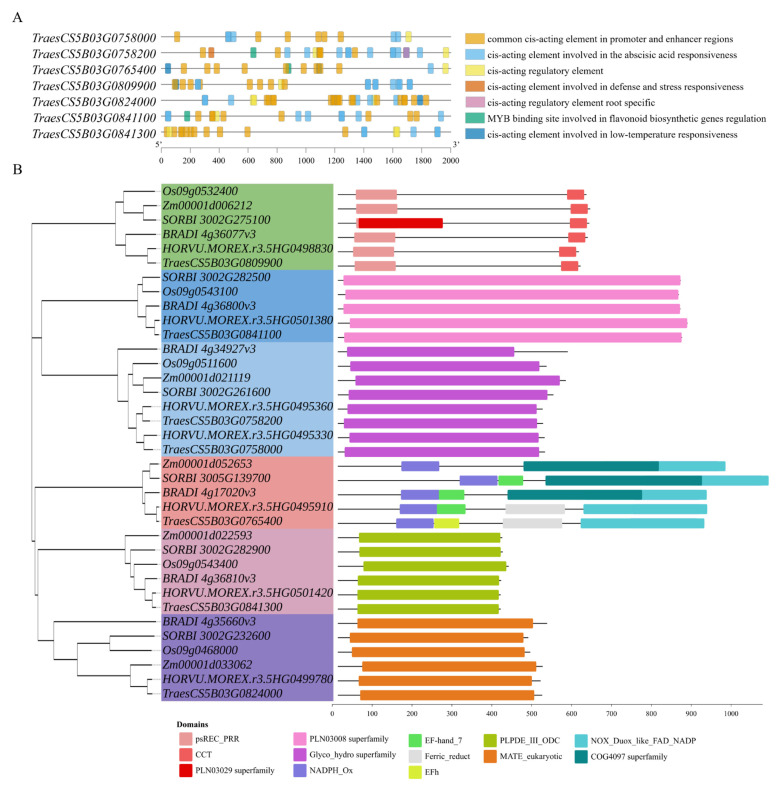
Analysis of cis-acting elements in the promoter (**A**). Phylogenetic tree, and conserved domains of homologous genes from related species (**B**).

Following KASP marker validation, seven possible candidate genes on chromosome 5B were confirmed to reside within the verified target QTL intervals. To explore their transcriptional regulatory logic, cis-acting elements in their promoter regions were analyzed, and seven categories of core regulatory elements related to low-temperature and root responses were identified, including low-temperature-responsive and defense/stress-responsive cis-acting elements (Figure 7A).

Subsequently, to investigate the evolutionary conservation and interspecific divergence characteristics of the seven aforementioned low-temperature tolerance candidate genes, we selected homologous gene sequences from five closely related species, including *O. sativa*, *Z. mays*, *Hordeum vulgare*, *Brachypodium distachyon* and *Sorghum bicolor*, to construct a phylogenetic tree of homologous genes. Based on the shared conserved domain information, 6 major evolutionary clades were identified, and 13 conserved domains were detected (Figure 7B).

## 4. Discussion

Low-temperature damage is a critical factor limiting the geographical distribution and yield of wheat. Frost injury induces a series of adaptive changes during wheat growth, which can be assessed through both external plant characteristics (e.g., dead seedling rate, dead tiller rate) and internal physiological parameters (e.g., cell membrane permeability, electrical conductivity, reactive oxygen species (ROS) levels) [41,42]. Field evaluation focuses on external traits to study wheat cold resistance, while winter hardness degree classification serves as the most direct method for assessing a variety’s cold tolerance [43]. You et al. [44] investigated cold hardness of 71 wheat varieties grown in the Yellow-Huai-Hai River valley region and found that cold hardiness was correlated with other stress tolerances. The frost tolerance of 491 wheat accessions was quantitatively assessed by the degree of frost damage to leaves and stems; combined with GWAS, a total of 107 QTL were identified [45]. However, field identification is significantly influenced by environmental factors, necessitating the design of multi-year and multi-site repeated experiments. Moreover, in warm winter years, the severity of cold damage may be insufficient for classification. Therefore, artificial simulation of low-temperature environments is more feasible and reproducible for evaluating plant cold resistance. Zhao et al. [46] used artificial freezing to investigate the relative conductivity and freezing survival rate of 209 wheat varieties at seedling stage, and several cold resistance varieties were obtained. Ju et al. [47] identified 21 QTLs associated with cell membrane permeability of leaf treated with low temperature using 168 double haploid lines derived from the cross Huapei 3× Yumai 57. Most of these investigations focused on the aboveground parts of wheat; however, aboveground and belowground organs exhibit distinct response mechanisms, even at the molecular level [10], and root growth is generally more sensitive to temperature than aboveground plant parts [13]. Low temperature physiologically reduces the supply of water and nutrients from roots to leaves, leading to cellular water deficit and osmotic stress [48]. Root growth in wheat seedlings is closely linked to environmental conditions. In this study, low temperatures severely inhibited root development during the seedling stage, with consistent yet different patterns across the two different cultivation systems. While MRL, TRL, RD, and RA decreased in both NS and VC systems when they suffered cold conditions, the reduction was more pronounced in NS. This phenomenon may be attributed to the higher thermal conductivity of the nutrient solution compared to vermiculite, which weakens the latter’s insulating properties. Consequently, improper winter tillage practices that loosen soil structure expose roots to air, suppressing growth and adversely affecting wheat development in later stages [49].

Linkage analysis has been widely applied to identify loci associated with wheat cold tolerance [16,17]. The seedling cold tolerance coefficient serves as a comprehensive indicator of the impact of seedling cold on wheat growth. In this study, a total of 9 QTLs related to seedling cold tolerance coefficient were detected, distributed across chromosomes 1B, 2B, 3B, 3D, 4A, 5A, 5B and 7D. The identified QTL *QCi.saw-1B.1* is located on chromosome 1B at 4.52–15.75 Mb, overlapping with the previously reported QTL *QFT.ahau-1B* (5.63–9.60 Mb), which contains the FT reporter gene *ADC* (9.29 Mb) [6]. *QCi.saw-1B.2* is located on chromosome 1B at 58.75–59.60 Mb, adjacent with the previously reported QTL *qCT1B.1* (peak marker located at 39.85 Mb) [40]. *QCi.saw-5B* is located on chromosome 5B at 476.63–523.19 Mb, overlapping with the previously reported QTLs *qCT5B.3* (peak marker located at 502.02 Mb) [40], and *QTL_5B* (489.84–490.76 Mb) [15]. The remaining six loci have not been reported in other studies and are presumed to be novel root cold tolerance loci.

The DEGs following low-temperature treatment were enriched in biological processes related to plant growth and development, sugar metabolism, lipid metabolism, and environmental adaptation, with molecular processes primarily concentrated in enzyme activity-related pathways. Previous studies have demonstrated that trehalose metabolism regulates plant growth, development, and reproduction [50]. Additionally, many DEGs are involved in sugar metabolism processes, indicating that energy supply under low temperatures is particularly critical for wheat’s adaptation to cold conditions. KEGG pathway analysis revealed that these EDGs were enriched in pathways related to flavonoid synthesis, glutathione metabolism, and fatty acid elongation. The phenylpropanol pathway is a critical metabolic route for plants to cope with both abiotic and biotic stresses, with flavonoid metabolism being a key branch of phenylpropanol metabolism [51]. Flavonoid accumulation enhances plant antioxidant capacity and cold tolerance [52]. Glutathione metabolism maintains the antioxidant properties of plant tissues and regulates redox-sensitive signal transduction, with the glutathione metabolic pathway contributing to improved cold tolerance in plants [53,54]. Increased fatty acid content in plants also plays a significant role in mitigating cold damage [55]. Cold-tolerant varieties exhibit enriched KEGG pathways after cold stress, including the MAPK signaling pathway, as well as additional amino acid and lipid synthesis metabolic pathways that are activated under cold conditions [56].

In this study, we identified 13 putative genes in the QTL intervals for cold tolerance. The glycoside hydrolase family 1 (GH1) encompasses critical enzymes involved in plant hormone activation and signal transduction and plays essential roles in multiple physiological processes including plant growth and development. GH1 members also actively respond to various adverse environmental conditions such as cold, salinity, and drought stresses. Both *TraesCS5B03G0758000* and *TraesCS5B03G0758200* are annotated as β-glucosidases (BGLUs) in wheat, suggesting their potential involvement in cold stress adaptation through similar mechanisms observed in other plant species [57]. The phospholipase D (PLD) gene family constitutes a key group of enzymes involved in lipid-mediated signal transduction in plants and also plays a regulatory role in rice growth and development [58]. Previous studies have demonstrated that *PLD* genes actively participate in the regulation of plant responses to various abiotic stresses, including salinity, cold, and drought [59]. As a homologous gene of *OsPLD1* in rice and *ZmPLD15* in maize, *TraesCS5B03G0841100* in wheat is predicted to possess similar functions in mediating plant cold stress tolerance. The WRKY transcription factors are critical for plant growth, development, and adaptation to stress [60,61]; the expression level of *OsWRKY24* was up-regulated under low temperature [62,63], and it is recognized as potential candidate gene to influence the cold sensitivity of “Towada” and “ZL31” [64]. In this study, its orthologue gene *TraesCS3B03G0951200* is also induced and actively expressed under low-temperature conditions, indicating its involvement in the response to cold stress. Further investigation is needed to elucidate the specific regulatory mechanisms among these genes in the future.

## 5. Conclusions

A total of nine quantitative trait loci associated with the low-temperature tolerance coefficient were identified, and six of them have not been reported in previous studies; among them, *QCi.saw-2B* and *QCi.saw-5B* were validated in a natural population. Integrating transcriptome profiling and QTL mapping enabled the screening of 13 candidate genes responsible for regulating root low-temperature tolerance. These findings provide a valuable theoretical foundation and important genetic resources for gene cloning, molecular marker development, and low-temperature stress-resistant breeding in wheat.

## Figures and Tables

**Table 1:** Analysis of root traits at the seedling stage.

Traits	Nutrient Solution Culture					Vermiculite Cultivation				
	6 °C		24 °C		CI	6 °C		24 °C		CI
	Mean ± SD	CV (%)	Mean ± SD	CV (%)	Mean ± SD	Mean ± SD	CV (%)	Mean ± SD	CV (%)	Mean ± SD
MRL (cm)	7.40 ± 1.42 **	19.17	16.09 ± 3.01	18.70	0.47 ± 0.11	13.77 ± 1.54 **	11.20	17.37 ± 1.59	9.18	0.80 ± 0.09
TRL (cm)	21.24 ± 5.29 **	24.88	48.62 ± 11.09	22.81	0.46 ± 0.17	40.79 ± 5.98 **	14.67	62.76 ± 8.89	14.16	0.66 ± 0.11
RA (cm^2^)	2.94 ± 0.75 **	25.49	5.81 ± 1.31	22.60	0.54 ± 0.22	5.47 ± 0.94 **	17.14	8.23 ± 1.14	13.77	0.67 ± 0.12
RV (cm)	0.03 ± 0.01 **	28.00	0.06 ± 0.01	24.83	0.64 ± 0.29	0.06 ± 0.01 **	20.79	0.09 ± 0.01	16.37	0.69 ± 0.14
RD (mm)	0.44 ± 0.06 **	12.95	0.41 ± 0.06	14.88	1.09 ± 0.22	0.40 ± 0.04 **	9.61	0.35 ± 0.05	13.20	1.16 ± 1.21

** indicates a significant difference at the 0.01 probability level.

**Table 2:** Stable QTLs for wheat seedling traits detected in RILs.

Trait	QTL	Env	Chr.	LOD	PVE (%)	Add	Peak Marker	Genetic Interval (cM)	Physical Interval (Mb)
MRL	*QMrl.saw-2A*	24VC	2A	7.43	14.06	−0.61	*RAC875_c18928_529*	445.2–486.38	762.51–793.15
	*QMrl.saw-3B*	6VC	3B	3.50	6.31	−0.40	*BS00065978_51*	291.6–296.14	611.18–636.45
	*QMrl.saw-4B*	6NS	4B	3.40	8.34	−0.41	*IACX2314*	85.66–85.66	612.25–612.85
	*QMrl.saw-5A*	6VC, 24VC	5A	7.39	14.11	−0.62	*Excalibur_c7556_381*	25.74–118.1	538.00–574.75
	*QMrl.saw-5B.1*	24NS	5B	4.38	10.49	1.67	*Kukri_c9073_1450*	264.95–277.51	457.59–479.32
	*QMrl.saw-5B.2*	6NS	5B	3.01	7.34	−0.39	*BS00105054_51*	355.73–365.33	557.11–566.97
	*QMrl.saw-6B*	24VC	6B	4.39	7.95	0.46	*wsnp_Ex_c5075_9013594*	55.35–61.55	207.93–288.80
	*QMrl.saw-7B.1*	6VC	7B	3.63	6.56	−0.42	*Ku_c9598_2131*	61.9–63.88	153.18–181.04
	*QMrl.saw-7B.2*	24NS	7B	3.77	8.95	0.97	*Kukri_c87702_530*	118.85–128.18	678.83–689.52
	*QMrl.saw-7D*	24VC	7D	4.64	8.43	0.47	*RAC875_c3826_2443*	2.58–6.75	589.31–615.94
TRL	*QTrl.saw-1A*	6NS	1A	3.81	9.62	−1.71	*BS00075271_51*	19.84–23.55	585.82–586.91
	*QTrl.saw-2B*	24VC	2B	4.04	7.60	2.21	*Excalibur_c91034_141*	359.74–373.44	738.69–750.02
	*QTrl.saw-3A*	6NS	3A	3.84	9.58	−1.65	*RAC875_c15970_89*	9.7–17.94	699.77–701.24
	*QTrl.saw-4B*	6VC, 24VC	4B	3.39	5.94	1.50	*Tdurum_contig10302_187*	9.25–32.24	656.82–660.67
	*QTrl.saw-5A*	6VC, 24VC	5A	15.46	29.92	−3.36	*CAP11_c1685_149*	4.68–72.52	585.61–616.88
	*QTrl.saw-5B*	24NS	5B	3.33	8.33	3.36	*Kukri_rep_c103253_565*	264.02–269.07	470.21–479.20
	*QTrl.saw-6D*	6NS	6D	3.74	10.48	−1.82	*Excalibur_c24213_314*	1.01–5.41	352.36–352.36
	*QTrl.saw-7B*	6NS	7B	4.32	10.87	−1.84	*Kukri_c10659_1328*	6.48–12.19	740.04–744.26
	*QTrl.saw-7D*	24VC	7D	5.60	10.75	3.23	*RAC875_c3826_2443*	0.01–6.75	612.44–615.94
RA	*QRa.saw-2B*	24VC	2B	7.11	13.57	−0.51	*Excalibur_c5438_274*	360.74–434.12	738.69–775.17
	*QRa.saw-3A*	6NS	3A	6.21	15.55	−0.31	*RAC875_c15970_89*	2.61–22.94	679.25–701.24
	*QRa.saw-3B*	6VC	3B	4.79	8.71	−0.28	*wsnp_Ex_c34975_43204180*	218.93–239.96	507.51–558.51
	*QRa.saw-5A*	6VC, 24VC	5A	9.66	19.12	−0.54	*BobWhite_c15758_79*	0.01–16.67	585.00–647.36
RV	*QRv.saw-1B*	6NS	1B	6.42	15.44	0.01	*Ex_c67541_975*	199.43–213.59	448.14–520.53
	*QRv.saw-2B*	6NS	1B	4.46	10.37	0.00	*Jagger_c7242_85*	268.72–279.82	579.79–588.63
	*QRv.saw-2D*	24VC	2D	3.39	6.49	0.00	*RAC875_c25513_403*	453.55–440.11	634.3–654.61
	*QRv.saw-3A*	6NS	3A	4.69	11.70	0.00	*RAC875_c15970_89*	6.61–23.94	697.25–701.24
	*QRv.saw-3B*	6VC	3B	3.80	6.63	0.00	*Excalibur_c45968_83*	204–237.72	389.04–561.09
	*QRv.saw-3D*	6VC	3D	3.84	7.16	0.00	*D_GBF1XID01ETBRB_172*	28.8–29.8	596.09–596.09
	*QRv.saw-5A*	6VC, 24VC	5A	9.21	16.68	−0.01	*RFL_Contig316_572*	0.01–21.97	588.38–647.36
	*QRv.saw-6B.1*	24VC	6B	3.29	7.15	0.00	*RAC875_c68849_153*	14.37–26.69	26.63–32.33
	*QRv.saw-6B.2*	24NS	6B	3.49	8.60	0.00	*wsnp_Ex_c13896_21759905*	74.98–79.44	476.41–497.60
	*QRv.saw-6D*	24NS,	6D	3.22	7.90	0.00	*Kukri_c35951_337*	43.64–50.8	258.36–309.95
RD	*QRd.saw-3A*	24NS	3A	6.12	14.28	0.02	*BobWhite_c35093_176*	104.77–122.42	737.97–744.44
	*QRd.saw-3B*	24NS	3B	4.54	9.79	−0.02	*Tdurum_contig10107_580*	211.93–227.47	416.28–507.51
	*QRd.saw-6D*	24VC	6D	3.28	7.74	−0.01	*Ra_c106775_711*	43.08–44.65	309.95–309.95
	*QRd.saw-7D*	6VC	7D	3.25	6.89	0.01	*BS00035732_51*	29.82–32.15	516.03–517.22

**Table 3:** Stable CI QTLs detected in RILs.

QTL	Env	Chr.	LOD	PVE (%)	Add	Peak Marker	Genetic Interval (cM)	Physical Interval (Mb)	Known Genes/QTLs
*QCi.saw-1B.1*	VC-RA, VC-RV	1B	3.97	8.49	−0.04	*BS00022504_51*	1.01–62.32	4.52–15.75	*ADC* [6]
*QCi.saw-1B.2*	NS-MRL	1B	3.45	9.09	0.03	*Kukri_rep_c108299_221*	123.99–133.01	58.75–59.60	*qCT1B.1* [40]
*QCi.saw-2B*	VC-TRL	2B	3.47	7.75	0.03	*IACX11305*	425.85–445.21	765.28–781.58	
*QCi.saw-3B*	VC-MRL	3B	3.34	6.13	−0.02	*BS00065978_51*	291.6–294.89	611.18–636.25	
*QCi.saw-3D*	VC-RA, VC-RV	3D	5.76	12.54	0.05	*D_GBF1XID01ETBRB_172*	26.8–29.8	596.09–596.09	
*QCi.saw-4A*	NS-TRL	4A	3.97	11.02	0.06	*BS00062059_51*	142.77–154.6	634.74–640.25	
*QCi.saw-5A*	VC-RA, VC-RV	5A	4.79	9.64	−0.05	*wsnp_Ex_c356_698872*	217.77–228.65	36.32–37.06	
*QCi.saw-5B*	NS-MRL, NS-TRL	5B	5.23	13.94	−0.04	*BobWhite_s67247_276*	273.14–291.84	476.63–523.19	*qCT5B.3* [15,40]
*QCi.saw-7D*	VC-MRL	7D	4.31	8.84	−0.03	*D_contig18045_60*	2.57–4.74	612.44–615.94	

## Data Availability

The raw RNA-seq data have been deposited in PRJNA1449489 (https://www.ncbi.nlm.nih.gov/bioproject/PRJNA1449489, accessed on 6 April 2026). Further inquiries can be directed to the corresponding author.

## Appendix A

**Table A1:** Shanxi wheat variety information.

Number	Variety	Number	Variety	Number	Variety	Number	Variety
1	Jinmai 1	74	Linkang 11	147	Jintai 114	220	Yongmai 3
2	Jinmai 5	75	Zeyou 2	148	Chang 6794	221	Xin 6160
3	Jinmai 11	76	Donghei 1	149	Chang 7080	222	Changzhi 5608
4	Jinmai 12	77	Yunhei 28	150	Jintai 1310	223	Yunmai 766
5	Jinmai 16	78	Donghei 10	151	Chang 6990	224	Linxuan 2035
6	Jinmai 17	79	Linyou 2018	152	Zhongmai 247	225	Linxian 151
7	Jinmai 18	80	Linyou 2069	153	Linhan 6	226	Yunmai 2064
8	Jinmai 19	81	Jinchun 3	154	Lin Y8161	227	Shengmai 20
9	Jinmai 20	82	Jinchun 13	155	Zhonghan 110	228	Shengmai 104
10	Jinmai 21	83	Jinchun 15	156	Lvhan 1608	229	Jinmai 107
11	Jinmai 13	84	Jinmai 9	157	Lin Y8012	230	Pinyu 8155
12	Jinmai 22	85	Jinchun 14	158	Lvke 298	231	Donghei 1206
13	Jinmai 23	86	Jinchun 16	159	Jintai 141	232	Taizi 6336
14	Jinmai 24	87	Jinchun 17	160	Jintai 146	233	Hanyou 6
15	Jinmai 25	88	Taichun 3473	161	Tai 1305	234	Yun 85-24
16	Jinmai 27	89	Xiaoheimai 76	162	Tai 412	235	Jinmai 10
17	Jinmai 28	90	Jinchun 5	163	Yun 14 Guan 74	236	Jinmai 89
18	Jinmai 29	91	Yunhan 2335	164	Yaomai 16	237	Jinmai 6
19	Jinmai 31	92	Chang 6359	165	Shinong 086	238	Jingmai 6425
20	Jinmai 32	93	Changmai 5079	166	Xiangmai 23	239	Ziyou 5
21	Jinmai 30	94	Chang 6452	167	Linfen 6410	240	Zimai 8555
22	Jinmai 33	95	Chang 4640	168	Jinmai 101	241	Linmai 5311
23	Jinmai 35	96	Fenheimai 1831	169	Jinmai 102	242	Tai 615
24	Jinmai 36	97	Jinmai 78	170	Jintai 1515	243	Changmai 3809
25	Jinmai 37	98	Jinmai 79	171	NC206	244	Chang 7170
26	Jinmai 38	99	Jinmai 80	172	Zhongmai 175	245	Linnong 4357
27	Jinmai 39	100	Jinmai 81	173	Shunmai 1718	246	Jinmai 109
28	Jinmai 40	101	Fen 4846	174	Yunhan 1512	247	Womai 608
29	Jinmai 41	102	Fen 4439	175	Yunhan 139–2	248	Jinmai 105
30	Jinmai 42	103	Jinmai 82	176	Runmai 2	249	Jintai 1503
31	Jinmai 44	104	Linfen 8050	177	Xiangmai 8156	250	Dabaibai
32	Jinmai 43	105	Jinmai 83	178	Linhan 9	251	Xiaohongpi
33	Jinmai 45	106	Linfen 6510	179	Womai 323	252	Niuzhijia
34	Jinmai 46	107	Yunhan 20410	180	Jinmai 919	253	Honglimai
35	Jinmai 47	108	Changmai 6686	181	Jinmai 104	254	Jiangzhouhong
36	Jinmai 48	109	Chang 7016	182	Chang 6789	255	Jiahongmai
37	Jinmai 49	110	Jinmai 84	183	Lin Y7287	256	Niuzhijia
38	Jinmai 50	111	Jinmai 85	184	Yunhan 1411–2	257	Siyuemei
39	Jinmai 51	112	Jintai 9923	185	Yunhei 14207	258	Sanyuehuang
40	Jinmai 52	113	Jinmai 86	186	Yunhei 161	259	Baimangcao
41	Jinmai 53	114	Tai 5902	187	Yunnuo 32	260	Youmangdahongjing
42	Jinmai 54	115	Changmai 6135	188	Jinmai 106	261	Zhuchengqing
43	Jinmai 56	116	Jinmai 87	189	Longmai 1	262	Hongtumai
44	Jinmai 57	117	Yunhan 719	190	Jintai 1508	263	Baitumai
45	Jinmai 58	118	Jinmai 88	191	ZM148	264	Baitumai
46	Jinmai 59	119	Shannong 129	192	Taidong 2503	265	Hongtumai
47	Jinmai 60	120	Tai 13606	193	Chang 5638	266	Yequmai
48	Jinmai 61	121	Changmai 5973	194	Jintai 1510	267	Hongpidongmai
49	Jinmai 62	122	Chang 5222	195	Taichun 101	268	Baishangeda
50	Jinmai 63	123	Chang 6388	196	Hanke 4898	269	Hongyequ
51	Jinmai 65	124	Linyuan 8	197	Yunhan 618	270	Qisifeng
52	Jinmai 66	125	Changmai 251	198	Yunhan 115	271	Hongheshang
53	Jinmai 67	126	Chang 6197	199	Yunhan 102	272	Xianmai
54	Jinmai 68	127	Chang 8744	200	Yunhan 22–33	273	Penmai
55	Jinmai 70	128	Jinmai 90	201	Jinmai 8	274	Baikehong
56	Jinmai 71	129	Jinmai 91	202	Lumai 14	275	Hongxiaomai
57	Jinmai 72	130	Yunhan 805	203	Jinmai 77	276	Jinguoyin
58	Jinmai 73	131	Jinmai 92	204	Changmai 6789	277	Youbailan
59	Jinmai 74	132	Jintai 182	205	Yun 9805	278	Baixianmai
60	Jinmai 75	133	Chang 4853	206	Linhan 5325	279	Baigengmai
61	Linfeng 615	134	Jinmai 94	207	Chang 5804	280	Baihuomai
62	Yunyin 1	135	Jinmai 95	208	Yunhan 1818	281	Baisanyuehuang
63	Jinnong 207	136	Jinmai 96	209	Zhongmai 110	282	Yulanmai
64	Yunmai 1934	137	Jintai 102	210	Zhongyou 9507	283	Zimai
65	Chang 6878	138	Jinmai 97	211	Linfen 139	284	Datongxiaomai
66	Jintai 170	139	Jinmai 98	212	Zhongmai 349	285	Qisuimai
67	Linyou 145	140	Yunmai 218	213	Shunmai 612	286	Dahongmai
68	Linfen 138	141	Jinmai 99	214	Zeyou 1	287	Huoshaotou
69	Chang 6154	142	Yunhan 21–30	215	Changmai 3987	288	Dingxingzhaixiaomai
70	Hedong TX–006	143	Liangxing 67	216	Zhongyou 206	289	Baishanmai
71	Linfeng 3	144	Yunhan 137	217	Jinmai 100		
72	Jintai 65	145	Tai 113	218	Xinmai 296		
73	Linyuan 3158	146	Jinzuo 80	219	Lunxuan 167		

**Table A2:** KASP PCR thermal cycling conditions.

Stage	Temperature	Time	Number of Cycles for Each Stage
Initial denaturation	94 °C	15 min	1 cycle
Touchdown	94 °C	20 s	10 cycles (final annealing temperature of 55 °C)
	61 °C (drop-0.6 °C/per cycle)	60 s	
Amplification	94 °C	20 s	26 cycles
	55 °C	60 s	
Fluorescence acquisition	37 °C	1 min	1 cycle

**Table A3:** KASP marker information.

QTL	Marker Name	Primer Name	FAM Primer	HEX Primer	Common Primer
*QCi.saw-1B.1*	*BS00022504_51*	*QCi.saw-1B.1-KASP*	GAAGGTGACCAAGTTCATGCT GATGTGAGGCCATCCACAAT	GAAGGTCGGAGTCAACGGATT GATGTGAGGCCATCCACAAC	TTGAAGTACCTTGTACCAATTGTTT
*QCi.saw-1B.2*	*Kukri_rep_c108299_221*	*QCi.saw-1B.2-KASP*	GAAGGTGACCAAGTTCATGCT GATCAAGTGCTTTGGACACAAT	GAAGGTCGGAGTCAACGGATT GATCAAGTGCTTTGGACACAAC	AAAAACGCAGGATATGGTTGAG
*QCi.saw-2B*	*IACX11305*	*QCi.saw-2B-KASP*	GAAGGTGACCAAGTTCATGCT TGAAGCGAGGGACACCGT	GAAGGTCGGAGTCAACGGATT TGAAGCGAGGGACACCGC	CCGCCAGAGCATCATCCC
*QCi.saw-5A*	*wsnp_Ex_c356_698872*	*QCi.saw-5A-KASP*	GAAGGTGACCAAGTTCATGCT GAAGCGTTAAGACTGCCACA	GAAGGTCGGAGTCAACGGATT GAAGCGTTAAGACTGCCACG	CGTGATTGAATATGTAACGGCG
*QCi.saw-5B*	*BobWhite_s67247_276*	*QCi.saw-5B-KASP*	GAAGGTGACCAAGTTCATGCT GAATAAACCTGAGTGTGTGAATGTA	GAAGGTCGGAGTCAACGGATT GAATAAACCTGAGTGTGTGAATGTG	CGTACCGGTCTGAACATTCC
*QCi.saw-7D*	*D_contig18045_60*	*QCi.saw-7D-KASP*	GAAGGTGACCAAGTTCATGCT CTAGAGGTATGCGAAACTCGTT	GAAGGTCGGAGTCAACGGATT CTAGAGGTATGCGAAACTCGTC	GCCTGTACTGGAGATGATACGT

The underlined portions represent the fluorescent adapter sequences.

**Table A4:** Transcriptome sequencing data statistics.

Sample	ReadSum	BaseSum	Q20 Percentage (%)	Q30 Percentage (%)	GC (%)
CS_CK1	67,838,972	101,75,845,800	99.32	97.22	51.89
CS_CK2	67,445,802	10,116,870,300	99.33	97.29	50.06
CS_CK3	67,930,536	10,189,580,400	99.35	97.24	51.87
CR_CK1	63,050,376	9,457,556,400	99.3	97.19	50.24
CR_CK2	66,589,394	9,988,409,100	99.32	97.23	50.71
CR_CK3	73,296,646	10,994,496,900	99.31	97.23	50.52
CS_LT1	66,337,106	9,950,565,900	99.33	97.22	50.78
CS_LT2	80,408,464	12,061,269,600	99.27	97.1	52.8
CS_LT3	77,318,176	11,597,726,400	99.38	97.37	52.22
CR_LT1	78,379,094	11,756,864,100	99.4	97.48	50.56
CR_LT2	71,124,774	10,668,716,100	99.38	97.44	50.73
CR_LT3	86,765,258	13,014,788,700	99.35	97.37	53.32

**Table A5:** DEGs within QTL regions for CR.

QTL	Expression Regulation	Gens ID	Position	log2 FoldChange	*p* − *adj*	Description
*QCi.saw-1B.1*	UP	*TraesCS1B03G0036700*	9,183,314–9,184,082	2.17	4.94 × 10^−2^	defensin Tk-AMP-D2-like
		*TraesCS1B03G0036800*	9,188,088–9,202,583	1.78	4.56 × 10^−2^	methionine S-methyltransferase-like
		*TraesCS1B03G0037200*	9,239,611–9,240,396	2.79	6.10 × 10^−7^	defensin Tm-AMP-D1.2-like
	DOWN	*TraesCS1B03G0023900*	6,433,883–6,442,364	−1.25	2.64 × 10^−2^	Pm3-like disease resistance protein
		*TraesCS1B03G0035400*	8,744,706–8,761,253	−1.23	1.61 × 10^−2^	uncharacterized LOC123103182
		*TraesCS1B03G0046800*	11,097,738–11,101,755	−3.20	1.26 × 10^−4^	myb-related protein P-like
*QCi.saw-1B.2*	UP	*TraesCS1B03G0173600*	58,748,310–58,751,058	1.16	1.32 × 10^−2^	glycine-rich protein A3-like
*QCi.saw-2B*	UP	*TraesCS2B03G1409300*	763,263,155–763,264,871	1.86	2.21 × 10^−2^	metalloendoproteinase 1-MMP-like
		*TraesCS2B03G1423700*	767,901,180–767,902,334	2.10	2.57 × 10^−4^	cytosolic sulfotransferase 15-like
		*TraesCS2B03G1432400*	771,567,475–771,568,883	1.35	1.95 × 10^−2^	uncharacterized LOC123047285
*QCi.saw-3B*	UP	*TraesCS3B03G0951200*	612,132,065–612,134,652	2.24	1.28 × 10^−4^	WRKY transcription factor WRKY24-like
		*TraesCS3B03G0952700*	612,586,466–612,588,715	3.01	9.99 × 10^−4^	laccase-3
		*TraesCS3B03G0959100*	617,595,690–617,601,077	1.74	4.58 × 10^−2^	protein trichome birefringence-like 28
		*TraesCS3B03G0959800*	618,456,702–618,460,221	1.39	5.73 × 10^−3^	BAG family molecular chaperone regulator 4-like
		*TraesCS3B03G0961500*	618,861,251–618,866,296	1.69	7.70 × 10^−5^	cyclin-dependent kinase 11.2-like
		*TraesCS3B03G0964100*	620,511,463–620,513,912	1.27	3.24 × 10^−3^	DNA-3-methyladenine glycosylase-like
		*TraesCS3B03G0969500*	624,804,220–624,807,036	3.23	1.89 × 10^−3^	ABC transporter G family member 6-like
		*TraesCS3B03G0972900*	627,786,251–627,787,107	3.34	4.35 × 10^−3^	zinc finger protein ZAT11-like
		*TraesCS3B03G0975400*	630,720,805–630,723,797	3.31	2.73 × 10^−3^	heat shock cognate 70 kDa protein-like
*QCi.saw-4A*	UP	*TraesCS4A03G0909100*	640,910,991–640,912,110	2.76	8.52 × 10^−5^	uncharacterized LOC123082842
	DOWN	*TraesCS4A03G0897000*	636,133,539–636,138,327	−1.35	4.99 × 10^−6^	elongation factor 2
		*TraesCS4A03G0897300*	636,426,398–636,429,390	−2.20	3.62 × 10^−2^	calreticulin-like
*QCi.saw-5B*	UP	*TraesCS5B03G0747300*	476,824,009–476,829,820	1.09	4.45 × 10^−2^	probable protein S-acyltransferase 7
		*TraesCS5B03G0753400*	480,082829–480,085,064	1.09	1.90 × 10^−2^	uncharacterized protein At4g22758-like
		*TraesCS5B03G0758000*	481,742,460–481,748,414	1.89	1.04 × 10^−4^	beta-glucosidase 31-like
		*TraesCS5B03G0758200*	481,749,201–481,757,565	2.18	2.34 × 10^−4^	beta-glucosidase 31-like
		*TraesCS5B03G0765400*	485,288,712–485,291,893	2.45	1.29 × 10^−2^	respiratory burst oxidase homolog protein B-like
		*TraesCS5B03G0775200*	490,674,972–490,681,230	1.39	3.00 × 10^−2^	uncharacterized LOC123112720
		*TraesCS5B03G0784600*	494,219,624–494,220,425	5.64	4.69 × 10^−2^	RING-H2 finger protein ATL74-like
		*TraesCS5B03G0789800*	496,089,252–496,090,624	2.13	1.35 × 10^−2^	dehydration-responsive element-binding protein 1B
		*TraesCS5B03G0794700*	498,822,420–498,824,659	1.40	1.20 × 10^−2^	BAG family molecular chaperone regulator 3
		*TraesCS5B03G0804600*	504,924,584–504,928,616	1.00	3.33 × 10^−3^	B-box zinc finger protein 18-like
		*TraesCS5B03G0805000*	505,234,033–505,236,122	3.14	1.21 × 10^−2^	cytochrome P450 71A1-like
		*TraesCS5B03G0805600*	505,423,222–505,426,349	1.49	9.36 × 10^−3^	cytochrome b561 domain-containing protein At5g54830-like
		*TraesCS5B03G0809900*	508,769,139–508,773,029	4.05	1.11 × 10^−25^	two-component response regulator-like PRR95
		*TraesCS5B03G0810500*	509,273,023–509,276,322	2.57	1.46 × 10^−2^	MYB-like transcription factor ODO1
		*TraesCS5B03G0812500*	509,749,624–509,754,655	1.62	3.68 × 10^−2^	chaperone protein ClpD1, chloroplastic-like
		*TraesCS5B03G0824000*	513,924,809–513,930,075	2.68	7.86 × 10^−9^	protein DETOXIFICATION 40-like
		*TraesCS5B03G0832800*	518,349,477–518,354,198	1.05	2.64 × 10^−2^	3-dehydroquinate synthase, chloroplastic-like
		*TraesCS5B03G0838300*	521,021,356–521,025,215	1.15	1.71 × 10^−3^	aspartyl protease family protein 1-like
		*TraesCS5B03G0840700*	522,164,910–522,166,092	1.78	2.50 × 10^−2^	uncharacterized protein At1g76070-like
		*TraesCS5B03G0841100*	522,292,160–522,299,099	1.30	1.79 × 10^−2^	phospholipase D delta-like
		*TraesCS5B03G0841300*	522,375,810–522,377,272	2.21	2.52 × 10^−2^	ornithine decarboxylase-like
	DOWN	*TraesCS5B03G0819700*	511,733,311–511,736,671	−3.48	1.45 × 10^−3^	wall-associated receptor kinase 5-like
		*TraesCS5B03G0838100*	521,008,010–521,010,066	−1.55	4.74 × 10^−3^	putative UDP-rhamnose_rhamnosyltransferase 1
*QCi.saw-7D*	UP	*TraesCS7D03G1198900*	613,799,931–613,805,802	1.73	6.91 × 10^−4^	probable plastidic glucose transporter 3
		*TraesCS7D03G1200900*	614,259,268–614,267,071	1.13	3.65 × 10^−2^	probable sucrose-phosphate synthase 3
		*TraesCS7D03G1201000*	614,282,675–614,285,528	2.54	1.78 × 10^−3^	heptahelical transmembrane protein ADIPOR1-like

**Table A6:** DEGs within QTL regions for CS.

QTL	Expression Regulation	Gens ID	Position	log2 FoldChange	*p* − *adj*	Description
*QCi.saw-1B.1*	UP	*TraesCS1B03G0028800*	7,136,236–7,137,729	5.89	5.45 × 10^−7^	jacalin-related lectin 9-like
		*TraesCS1B03G0036700*	9,183,314–9,184,082	4.11	2.81 × 10^−7^	defensin Tk-AMP-D2-like
		*TraesCS1B03G0036800*	9,188,088–9,202,583	1.51	1.99 × 10^−2^	methionine S-methyltransferase-like
	DOWN	*TraesCS1B03G0040100*	9,782,831–9,784,602	−1.21	1.46 × 10^−2^	putative 12-oxophytodienoate reductase 5
		*TraesCS1B03G0042300*	10,227,059–10,228,066	−3.79	2.00 × 10^−3^	probable glutathione S-transferase GSTU6
		*TraesCS1B03G0061600*	15,868,602–15,870,408	−1.52	2.73 × 10^−2^	uncharacterized LOC123103821
*QCi.saw-2B*	DOWN	*TraesCS2B03G1444900*	775,782,837–775,786,378	−1.56	4.73 × 10^−2^	vegetative cell wall protein gp1-like
		*TraesCS2B03G1446500*	776,537,600–776,541,483	−2.36	8.53 × 10^−4^	putative disease resistance RPP13-like protein 1
*QCi.saw-3B*	UP	*TraesCS3B03G0952700*	612,586,466–612,588,715	2.38	3.89 × 10^−5^	laccase-3
		*TraesCS3B03G0953000*	612,823,313–612,825,138	3.01	5.92 × 10^−5^	GDSL esterase/lipase At2g31540-like
		*TraesCS3B03G0969500*	624,804,220–624,807,036	2.18	3.50 × 10^−3^	ABC transporter G family member 6-like
*QCi.saw-3D*	DOWN	*TraesCS3D03G1118900*	596,648,466–596,651,491	−6.58	6.89 × 10^−3^	uncharacterized LOC123080798
*QCi.saw-4A*	DOWN	*TraesCS4A03G0897300*	636,426,398–636,429,390	−4.04	1.12 × 10^−5^	calreticulin-like
*QCi.saw-5B*	UP	*TraesCS5B03G0790100*	496,144,315–496,145,219	3.28	3.17 × 10^−2^	dehydration-responsive element-binding protein 1B
		*TraesCS5B03G0809900*	508,769,139–508,773,029	2.11	4.83 × 10^−4^	two-component response regulator-like PRR95
	DOWN	*TraesCS5B03G0777300*	491,517,922–491,519,542	−2.76	3.15 × 10^−2^	putrescine hydroxycinnamoyltransferase 3-like
		*TraesCS5B03G0793800*	497,312,446–497,313,439	−2.76	2.25 × 10^−2^	dehydration-responsive element-binding protein 1A
		*TraesCS5B03G0825200*	514,356,541–514,361,463	−3.71	3.81 × 10^−5^	5′-3′ exoribonuclease 2-like
*QCi.saw-7D*	UP	*TraesCS7D03G1201000*	614282,675–614,285,528	1.80	2.31 × 10^−3^	heptahelical transmembrane protein ADIPOR1-like
	DOWN	*TraesCS7D03G1199200*	613,890,835–613,891,492	−3.30	1.49 × 10^−2^	probable calcium-binding protein CML25/26
